# Pituitary dysfunction with eosinophilic granulomatosis with polyangiitis presenting with diabetes insipidus: a case report and review of the literature

**DOI:** 10.3389/fimmu.2025.1557555

**Published:** 2025-06-06

**Authors:** Aifei Zhang, Xiaoxia Liu, Pengjia Wu, Longyan Qin, Na Li, Yi Yin, Jiashun Zeng

**Affiliations:** ^1^ Department of Rheumatology and Immunology, The Affiliated Hospital of Guizhou Medical University, Guiyang, China; ^2^ Department of Radiology, The Affiliated Hospital of Guizhou Medical University, Guiyang, China

**Keywords:** eosinophilic granulomatosis with polyangiitis, central nervous system, hypophysitis, central diabetes insipidus, mepolizumab

## Abstract

Central diabetes insipidus secondary to hypophysitis in eosinophilic granulomatosis with polyangiitis (EGPA) is very rare, and this article summarizes one case reported from our site as well as two previously reported patients with EGPA, both of whom had central diabetes insipidus, suggesting that new, rare organ involvement (pituitary gland) may occur in the early stages of EGPA or years later. The aim is to improve our understanding of central diabetes insipidus caused by EGPA involving the pituitary gland and avoid misdiagnosis or missed diagnosis.

## Introduction

Eosinophilic granulomatosis with polyangiitis (EGPA) is a chronic inflammatory disease characterized by multisystem manifestations. Common manifestations include asthma, chronic rhinosinusitis with or without polyposis, pulmonary involvement, and peripheral blood eosinophilia (1). EGPA mainly affects the respiratory tract, lung, peripheral nervous system, heart, gastrointestinal tract, and skin, but central nervous system (CNS) involvement is rare. CNS involvement is mainly characterized by brain hemorrhage, brain infarction, cranial nerve palsy, optic neuritis, etc., and central diabetes insipidus (CDI) secondary to involvement of the pituitary gland is even rarer; only a few cases have been reported. In this study, we documented a patient with EGPA who presented to the Department of Endocrinology at our site with symptoms of polydipsia and polyuria and was considered to have CDI, which was definitively diagnosed as CDI secondary to EGPA involving the pituitary gland through a consultation in our department. We searched PubMed, Web of Science, Embase, Cochrane Library, and Medline databases, and there were two cases of patients with EGPA with CDI. We made a systematic analysis and review of the literature combined with the cases in our site, with the purpose of enhancing the understanding of CDI caused by EGPA involving the pituitary gland, reducing misdiagnosis or missed diagnosis, as well as exploring the treatment methods.

## Case report

**Figure 1:**
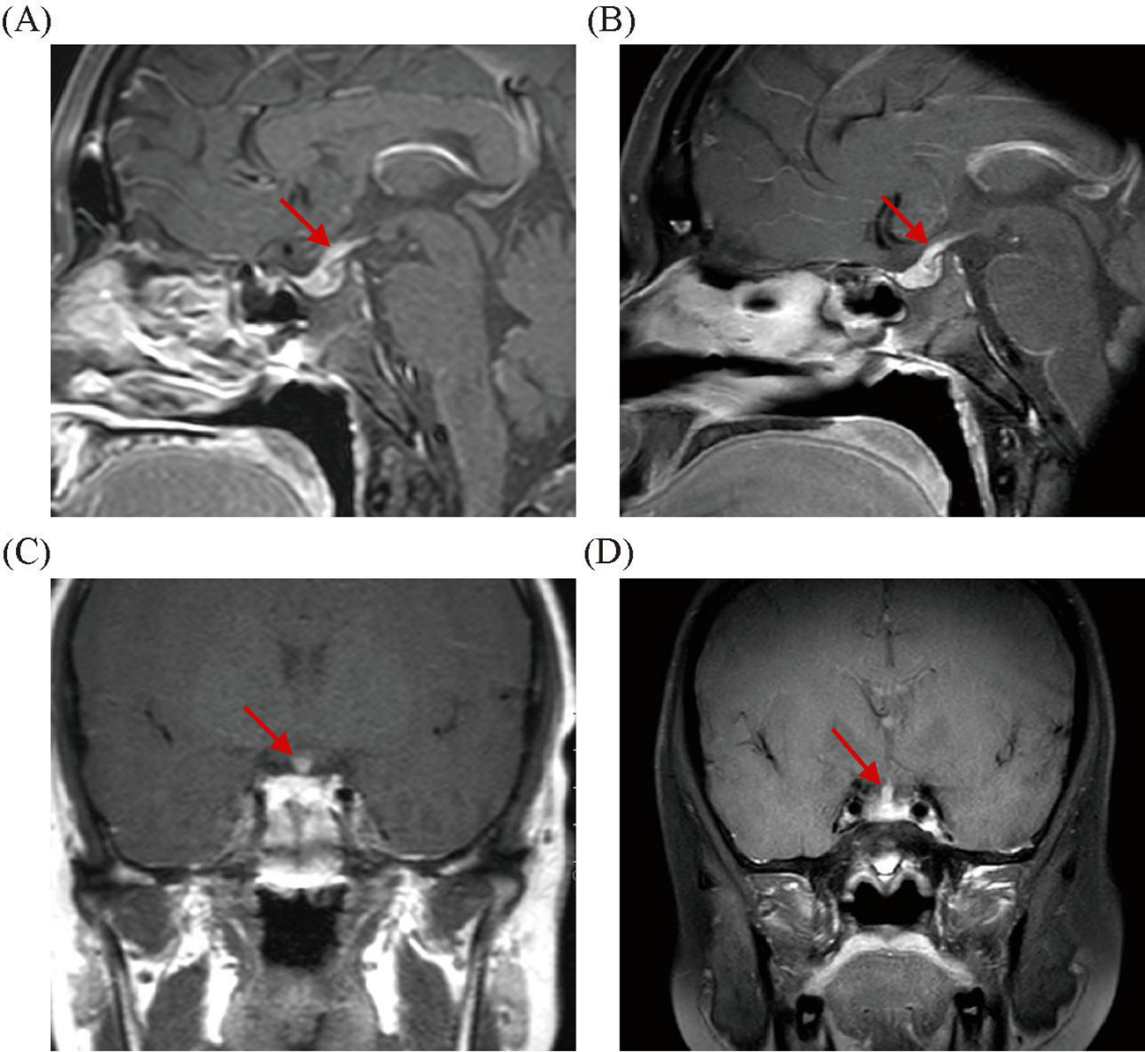
The pretherapeutic scan shows markedly increased volume of the pituitary gland **(A, C)**. The 6-month follow-up images showed a reduction in pituitary volume **(B, D)**.

A 19-year-old male patient was admitted to the Department of Endocrinology due to polydipsia and polyuria for 1 month, which was mainly manifested as polydipsia without obvious inducement 1 month ago, with a daily water intake of approximately 8,000–9,000 mL and a daily urine volume of approximately 10 L, accompanied by nausea, impaired appetite, occasional palpitation, and a weight loss of 7 kg. The patient had a history of bronchial asthma for more than 4 years and was currently treated with budesonide and formoterol fumarate powder for inhalation; he had a history of secretory otitis media for more than 4 years, which could be relieved by intermittent oral prednisone acetate; he had previously undergone double insertion of grommet, and still had intermittent discharge of pus from both ears with hearing decreased after surgery; he was diagnosed with Henoch–Schonlein purpura more than 3 years ago and was improving as a result of hormone therapy. Physical examination showed stable vital signs, clear consciousness, moon face, no cranial deformity, a little discharge and pus in the external auditory canal of both ears, scattered striae purple in both lower limbs, no abnormality in cardiopulmonary and abdominal examination, and no abnormality in neurological examination. Laboratory tests were as follows: absolute eosinophil count (ESO#), 1.46×10^9^/L (reference: 0.02–0.05×10^9^/L); eosinophil percentage (ESO%), 19.70%; blood osmolality, 286 mOsm/L; blood potassium, 4.07 mmol/L; blood sodium, 143.98 mmol/L; blood phosphorus, 0.640 mmol/L; fasting blood glucose, 4.77 mmol/L; blood creatinine, 49 μmol/L; C-peptide (fasting), 468.00 pmol/L; insulin (fasting) INS, 3.73 μIU/m; prolactin (PRL), 519.00 mIU/L (reference: 81–483 mIU/L); adrenocorticotropic hormone (ACTH), 7.37 pg/mL (reference: 7.20–63.30 pg/mL); thyroid-stimulating hormone (TSH), 4.20 mIU/L (reference: 0.510–4.30 mIU/L); luteinizing hormone (LH), 4.22 IU/L (reference: 1.70–8.60 IU/L); follicle-stimulating hormone (FSH), 3.44 IU/L (reference: 1.50–12.40 IU/L); immunoglobulin IgG4, 25 mg/dL; blood sedimentation rate, 2 mm/h; C-reactive protein, 0.25 mg/L; and urine specific gravity (SG), 1.005. The results of the vasopressin water deprivation test revealed CDI. Both anti-myeloperoxidase (anti-MPO) and anti-proteinase 3 (anti-PR3) antibodies tested negative. ANA antibody profile and cardiolipin antibodies showed no abnormalities. The positron emission tomography–computed tomography (PET-CT) scan revealed bilateral ethmoid sinusitis, bilateral maxillary sinusitis, and bilateral mastoiditis of the middle ear, with no evidence of neoplastic lesions detected elsewhere in the body. Cranial magnetic resonance imaging (MRI) scan revealed bilateral highmoritis, ethmoidal sinusitis, and bilateral otomastoiditis; dynamic contrast-enhanced MRI scan of the pituitary gland revealed slightly thickened and significantly enhanced pituitary stalk, approximately 4 mm in diameter, considering hypophysitis (**Figures 1A, C**). The diagnosis was CDI due to a pituitary lesion. After antidiuretic therapy with desmopressin 0.05 g bid, polydipsia and polyuria symptoms were relieved, water intake and urine volume were significantly reduced, but still did not return to normal levels, 24-h water intake: 5,500 mL, and 24-h urine volume: 6,000 mL. We considered the patient’s pituitary lesion was due to secondary factors. Our departmental consultation combined the patient’s history of peripheral blood eosinophilia, asthma, binaural otitis media (with granuloma formation), and previous hormone sensitivity, and the diagnosis of EGPA was made based on the ACR/EULAR EGPA classification criteria of 2022, with a total score up to 8, and the Birmingham Vasculitis Activity Score (BVAS) was 10. Remission-induction treatment of prednisone acetate 1 mg/kg combined with cyclophosphamide was administered for immunosuppression, but cyclophosphamide was discontinued after the patient developed a generalized skin rash (severe allergic reaction) due to cyclophosphamide, and was replaced with rituximab 375 mg/m^2^ (once a week, four times in total). After treatment, the patient’s symptoms of polydipsia, polyuria, asthma, and binaural otitis media were relieved, with a 24-h water intake of 2,500–3,000 mL and a 24-h urine volume of 3,000 mL. Blood routine showed absolute eosinophil count, 0.31×10^9^/L; blood osmolarity, 280 mOsm/L; blood PRL, 257.80 mIU/L; and urine SG, 1.015; dynamic contrast-enhanced MRI scan of the pituitary gland revealed decreased absorption of hypophysitis lesions (**Figures 1B, D**). During the subsequent 1-year follow-up, the patient used hormone combined with methotrexate to maintain the remission therapy. However, when the hormone dose was reduced to <15 mg/day, the symptoms of polydipsia, polyuria, asthma, and otitis media were aggravated, and eosinophil values were increased. According to the patient’s condition, mepolizumab (300 mg, once a month) was added to the treatment. After treatment, the above symptoms were relieved, and peripheral eosinophil values remained normal. After five doses of mepolizumab, the patient’s condition was stable when the hormone was reduced to 7.5 mg/day. The 24-h water intake was 2,000 mL, and 24-h urine volume was 1,500–2,000 mL. The absolute eosinophil value was 0.05×10^9^/L, eosinophil percentage was 0.2%, blood osmolality was 290 mOsm/L, blood PRL was 345.20 mIU/L, and urine SG was 1.015; the dynamic contrast-enhanced MRI scan of the pituitary gland showed that hypophysitis lesions were decreased, and bilateral highmoritis, ethmoidal sinusitis, and sphenoidal sinusitis were improving than before. BVAS was 2 and achieved disease relief.

## Review of the literature

We conducted a literature review by searching PubMed, Web of Science, Embase, Cochrane Library, and Medline databases using the search terms “vasculitis OR EGPA” AND “hypophysitis (or Diabetes insipidus)”, and the inclusion criteria for selecting articles were as follows: (1) definite diagnosis of EGPA and CDI, and (2) study subjects were humans. Exclusion criteria included the following: (1) duplicate data from the same site, and (2) review or meta-analysis articles. A total of 29 studies published between 1980 and 2024 on antineutrophil cytoplasmic antibody (ANCA)-associated vasculitis (AAV) complicated by CDI were identified. Of these, 27 were excluded because they focused on CDI secondary to microscopic polyangiitis (MPA) or granulomatosis with polyangiitis (GPA). Thus, there were a total of two articles on EGPA with CDI manifestations (2, 3), coupled with one case presented in this study, giving a total of three patients with EGPA. Among these cases, there were two male patients and one female patient. In all three patients, after treatment for EGPA, not only was vasculitis stable, but symptoms of CDI were also gradually improving (**Table 1**).

**Table 1:** Clinical data of patients with central diabetes insipidus secondary to EGPA.

Item	Case 1^2^	Case 2^3^	Case3
Age (years)	54	51	19
Sex	Male	Female	Male
Respiratory system	Cough Polydipsia Polyuria Allergic rhinitis Shortness of breath	Sinusitis Mastoiditis Polyuria Polydipsia Bronchial asthma Mononeuritis multiplex	Polyuria Polydipsia Secretory otitis media Bronchial asthma
Eos%	5% (normal 0.5%–5%)	30.8 (normal 0.5%–5%)	19.70% (normal 0.5$–5%)
Eos#	560×10^6^/L (normal 50–300×10^6^/L)	—	1.46×10^9^/L (normal 0.02–0.52×10^6^/L)
Serum osmolality	—	—	286 mOsm/L
MPO ANCA	Positive	Positive	Negative
Brain MRI	Hypophysitis	Hypophysitis	Hypophysitis
Treatment	PSL	PSL, CPM, RTX, and MPZ	PSL, CPM, RTX, and MPZ
Outcome	Improved	Improved	Improved

PSL, prednisolone; CPM, cyclophosphamide; RTX, rituximab; MPZ, mepolizumab.

## Discussion

EGPA is a rare systemic necrotizing small vessel vasculitis, belonging to the group of AAV, and ANCA is positive in one-third of cases with specificity for MPO (4). However, the disease manifestations of EGPA are more complex than those of other AAV because eosinophils are one of the key elements in the diagnosis and pathogenesis of EGPA, and tissue damage may originate from either necrotizing vasculitis or eosinophil proliferation and activation. EGPA is diagnosed at an average age of 40 years old, and is relatively rare in children and adolescents, and when it occurs in this age group, its course appears to be more aggressive, often with significant pulmonary and cardiovascular manifestations (5). EGPA can affect any organ system, and severe cases can lead to CNS complications (6). A retrospective study of 338 patients with EGPA found that the average age at diagnosis in these patients was 50.3 ± 15.7 years old, and there was no difference in morbidity between male and female, with clinical manifestations including asthma (91.1%), peripheral neuropathy (51.4%), ear, nose, and throat (ENT) symptoms (48.0%), skin disorder (39.7%), pulmonary infiltration (38.6%), cardiac involvement (16.4%), and CNS (5.2%) (7). Analysis based on ANCA status revealed that ENT manifestations, peripheral nerve involvement, and renal disease were significantly more frequent in ANCA-positive patients, and conversely, the incidence of cardiomyopathy was significantly higher in ANCA-negative patients, but ANCA status did not differ in CNS manifestations (7, 8). The pathogenesis of ANCA-negative status in patients with EGPA remains incompletely understood. Current research suggests that ANCA-negative EGPA exhibits distinct immunopathological features compared to ANCA-positive EGPA, primarily involving a Th2-driven immune response and eosinophil-mediated inflammation rather than classic AAV. In ANCA-negative EGPA, elevated Th2 cytokine levels promote eosinophil differentiation, recruitment, and activation. Activated eosinophils release granular proteins and lipid mediators, directly contributing to tissue damage, resulting in clinical manifestations such as severe asthma, eosinophilic pneumonia, myocarditis, gastrointestinal involvement, or eosinophilic cardiomyopathy (9). Clinically, patients with ANCA-positive EGPA tend to experience more frequent vasculitis relapses, whereas ANCA-negative EGPA appears to be associated with higher mortality rates, likely attributable to increased cardiac involvement (7). In this article, there were two male patients and one female patient with EGPA who had respiratory symptoms and CNS involvement, but none had cardiac lesions. MRI scan showed changes of hypophysitis, and the ratio of ANCA positive to negative was 2:1. It seems that ANCA-positive status is associated with the involvement of pituitary gland, but due to the small sample size, more data are needed for verification.

The incidence of neurological involvement in EGPA ranges from 51% to 86%, and peripheral nervous system damage is usually predominant. In contrast, the CNS is rarely affected. CNS involvement may result from a combination of vasculitis and/or eosinophil-mediated injury, but the specific pathogenesis is unknown. The clinical manifestations of CNS involvement in EGPA varies widely, and all parts of the CNS may be affected, with ischemic cerebrovascular lesions and brain hemorrhage being the most common clinical manifestations reported in the literature. A retrospective cohort study included 88 cases of EGPA involving the CNS and found that 86% of patients with EGPA had CNS involvement at diagnosis, with predominant neuropathy manifested as ischemic cerebrovascular disease in 46 patients (52%); intracranial hemorrhage and/or subarachnoid hemorrhage in 21 patients (24%); visual acuity decreased in 28 patients (33%) (15 with optic neuritis, 9 with central retinal vein occlusion, and 4 with cortical blindness); and cranial nerve palsy in 18 patients (21%) (6), but no cases involving pituitary lesion were reported in these 88 EGPA cases. Another Chinese population-based study similarly did not report cases of hypophysitis secondary to EGPA, which observed CNS involvement in 17.3% (19/110) of patients with EGPA, with ischemic lesions being the most common manifestation, accounting for 63.2% of 19 cases, followed by posterior reversible encephalopathy syndrome (36.8%), spinal cord involvement (15.8%), bulbar involvement (15.8%), and intracranial hemorrhage (15.8%), and multivariate analysis showed that age, disease course, and fever were potential independent risk factors for CNS involvement in EGPA (10).

Arginine vasopressin deficiency (AVP-D), formerly known as CDI, is due to hypothalamic or posterior pituitary damage resulting in absent or subnormal AVP secretion, and is rare, with a prevalence of approximately 1/25,000 (11). The majority of AVP-D cases are acquired, with the most common causes including autoimmune neurohypophysitis, primary or secondary tumors, infiltrative diseases (such as Langerhans cell histiocytosis and sarcoidosis), neurosurgical interventions, and head trauma (**Table 2**). CDI has been reported to occur rarely (1%–3%) in granulomatous polyangiitis (GPA) (12, 13) and even more rarely in patients with EGPA. In this article, three patients with EGPA presented with CDI-related symptoms, abnormal laboratory parameters, and changes in pituitary imaging, which were relieved after treatment of EGPA, and pituitary MRI scan revealed the disappearance of pituitary inflammatory lesions, especially in one patient with EGPA who had increased prolactin levels due to pituitary stalk inflammation compressing the capillaries in the hypophyseal portal system, reducing the delivery of dopamine to the lactotroph cells (14); not only was pituitary inflammation absorbed, but prolactin levels returned to normal after treatment. In this article, one patient developed CDI many years after the diagnosis of EGPA, and the other two patients were definitely diagnosed with EGPA after presentation with CDI, and these cases suggest that new, rare organ involvement, such as the pituitary gland, may occur in the early stages of EGPA or years later. Because CDI secondary to EGPA involving hypophysitis is very rare, and the incidence is still unclear, it is easy to lead to misdiagnosis or delayed diagnosis in clinical work. Therefore, when patients with EGPA have polydipsia and polyuria symptoms during treatment or follow-up, the examination should be actively perfected, which is helpful for early identification and avoids missed diagnosis and misdiagnosis; at the same time, screening for rare diseases such as EGPA is recommended for patients with CDI. All three patients with CDI caused by EGPA showed changes of hypophysitis on imaging. In particular, dynamic contrast-enhanced MRI scan of the pituitary gland more accurately reflects the changes in the blood supply of the pituitary gland, and is helpful in recognizing early lesions as well as the nature and extent of the lesions. PET-CT can serve as a complementary tool to MRI in the diagnosis of hypophysitis, providing value in detecting pituitary size changes, assessing pituitary metabolic activity, differentiating pituitary adenoma from hypophysitis, and aiding in the identification of neoplastic lesions.

**Table 2:** Acquired etiologies of central diabetes insipidus.

Acquired etiology	Common clinical manifestations	Key diagnostic tests	Exclusion criteria in this case
Sarcoidosis	Cough, dyspnea, chest pain, skin nodules	Chest CT (hilar lymphadenopathy, lung parenchymal involvement), tissue biopsy (non-caseating granulomas)	Absence of relevant clinical manifestations; no significant findings on chest CT
Langerhans cell histiocytosis (LCH)	Bone pain, skin rash, pulmonary cysts	Whole-body bone scan/PET-CT, lesion biopsy (CD1a+, Langerin+ cells)	No relevant clinical manifestations; PET-CT shows no multifocal osteolytic lesions
IgG4-related disease	Pancreatic/lacrimal/salivary gland enlargement	Elevated serum IgG4; tissue biopsy (IgG4+ plasma cell infiltration)	Normal serum IgG4; no glandular enlargement on PET-CT
Meningitis/Encephalitis	Fever, meningeal irritation signs, altered consciousness	Cerebrospinal fluid analysis (elevated cell count, elevated protein); pathogen PCR/culture	No relevant clinical manifestations
Tuberculosis/Syphilis	Chronic polyuria with low-grade fever, weight loss, other organ tuberculosis involvement	Tuberculin test, CSF acid-fast staining, syphilis serology	No relevant clinical manifestations; negative TB/syphilis etiology/serology
Trauma/Surgery	History of head trauma or pituitary surgery	Pituitary MRI (pituitary stalk disruption or post-surgical changes)	No history of trauma/surgery
Pituitary adenoma/Craniopharyngioma	Headache, visual impairment, hypopituitarism	Pituitary MRI (sellar mass), full hormone panel (ACTH, TSH, etc.)	No tumor detected on pituitary MRI
Metastatic tumors (e.g., breast cancer, lung cancer)	Primary tumor symptoms (e.g., cough, bone pain)	Whole-body PET-CT, primary tumor marker screening	No evidence of primary tumor on PET-CT
Pituitary apoplexy	Sudden polyuria, severe headache, acute vision loss, altered consciousness	Pituitary MRI (signs of hemorrhage or infarction)	No pituitary hemorrhage/infarction on MRI
Drug/Toxin	History of specific medication use (e.g., lithium and rifampicin)	Drug withdrawal trial (symptom resolution), blood drug level testing	No history of specific medication use

Data on the treatment management and prognosis of CNS involvement in patients with EGPA are also scarce, and evidence-based guidelines for the diagnosis and management of EGPA published in *Nature*’s subjournal *Nature Reviews Rheumatology* in 2023 (15) recommend that remission-induction treatment should be tailored on the basis of clinical manifestations with prognostic relevance, and Five-Factor Score (FFS) should be considered when choosing remission-induction strategies to assess the presence of adverse prognostic factors. Studies have shown that CNS involvement has a poor prognostic impact on EGPA (16), and although the revised FFS (17) no longer contains CNS involvement, most studies using FFS as the treatment decision have cited its original version (16), and there is therefore a need to give higher-intensity treatment to such patients, and remission-induction treatment of high-dose glucocorticoids combined with cyclophosphamide or rituximab is an important treatment regimen, but cyclophosphamide is more affordable and more likely to penetrate the blood–brain barrier compared to rituximab (18). In addition, intrathecal injection of methotrexate 10 mg and dexamethasone 20 mg has been found to achieve beneficial effects in patients with refractory EGPA with CNS involvement (10). The IL-5 inhibitor mepolizumab combined with glucocorticoids is recommended to induce remission in patients with relapsed and refractory EGPA without organ damage or life-threatening manifestations, and can also be used for maintenance therapy, but evidence-based medical data are currently lacking to demonstrate the effectiveness of mepolizumab in patients with EGPA with CNS involvement. Three cases of EGPA reported in our study were treated with glucocorticoids, two patients were treated with cyclophosphamide and rituximab, and clinical symptoms associated with diabetes insipidus were relieved with mepolizumab. Desmopressin could be tapered, and MRI scan showed absorption of pituitary inflammation. These two patients are the first cases describing the use of mepolizumab for the treatment of pituitary dysfunction caused by EGPA. Patients with EGPA in our site had multiple recurrences of clinical symptoms after remission-induction treatment of hormone combined with rituximab, requiring an increase in glucocorticoid dose to control the disease. After the addition of mepolizumab, the disease was gradually stabilized, and respiratory symptoms and diabetes insipidus symptoms were relieved, while the hormone dosage could be gradually reduced, suggesting that the use of traditional treatment regimens combined with mepolizumab seems to be a promising therapeutic measure for patients with pituitary dysfunction due to EGPA involving hypophysitis or for patients with EGPA involving the CNS.

## Conclusions

CDI secondary to hypophysitis in EGPA is very rare, and screening for autoimmune diseases, including rare disorders such as EGPA, is recommended for patients with CDI.

## Data Availability

The original contributions presented in the study are included in the article/supplementary material. Further inquiries can be directed to the corresponding author.
